# Detection of cytokines in cervicovaginal lavage in HIV-infected women and its association with high-risk human papillomavirus

**DOI:** 10.3389/fimmu.2024.1416204

**Published:** 2024-06-28

**Authors:** Sandra Schindler, Eduardo Netto, Felice Deminco, Camila A. Figueiredo, Candace Machado de Andrade, Amanda Rosa Alves, Carlos Brites

**Affiliations:** ^1^ Programa de Pós-graduação em Medicina e Saúde (PPgMS), Universidade Federal da Bahia, Salvador, Brazil; ^2^ Faculdade de Medicina, Universidade Federal da Bahia, Salvador, Brazil; ^3^ Laboratório de Pesquisa em Infectologia (LAPI), Hospital Universitário Prof. Edgard Santos (HUPES), Universidade Federal da Bahia, Salvador, Brazil; ^4^ Instituto de Ciências da Saúde, Universidade Federal da Bahia, Salvador, Brazil

**Keywords:** HIV, AIDS, human papillomavirus, cervicovaginal cytokines, inflammation, IL-10, cervical cancer

## Abstract

**Background:**

Women living with HIV/AIDS (WLHA) have an increased prevalence of high-risk HPV infection (HR-HPV) and cervical intraepithelial neoplasia (CIN) and a greater risk of cervical cancer despite access to a new generation of antiretroviral therapy. The aim of this study is to evaluate the concentrations of different cytokines involved in the local immune response in WLHA, which is fundamental for understanding the pathogenesis of HPV-related cancer in this population.

**Methods:**

IL-1β, IL-2, IL-4, IL-6, IL-10, IFN-γ, TNF-α, IP-10, GM-CSF, and MIP-1α were investigated in the cervicovaginal lavage (CVL) of 106 WLHA attending at Hospital Universitario Professor Edgard Santos in Salvador, Bahia, Brazil, during the period December 2019 to April 2023 by Luminex^®^. All participants were also tested for *Chlamydia trachomatis* and *Neisseria gonorrhoeae* and underwent colposcopy, Pap smear, and Nugent score. HIV plasma viral load (VL) and CD4 cell count were performed for all WLHA.

**Results:**

In this study, 22.6% (24/106) of WLHA were infected with HR-HPV. A higher proportion of patients with HR-HPV (66.7%) had detectable levels of IL-10 than those negative ones (40.2%, *p* = 0.02). More premenopausal women had either IL-6 (51.4%) or IP-10 (58.3%) than those in menopausal status (26.5% for IL-6 and 32.4% for IP-10, *p* = 0.013 and *p* = 0.011, respectively). Vaginosis was negatively associated with detection of IP-10 (24.2% vs. 61.4%, *p* < 0.001) and INF-γ (39.4% vs. 68.6%, *p* = 0.005). A positive association was detected for IL-1β (66.7 vs. 37.1%, *p* = 0.005) and IL-10 (63.6% vs. 37.1%, *p* = 0.01). VL and CD4 were not associated with the studied cytokines.

**Conclusion:**

We demonstrated a positive association between IL-10 and HPV infection in CVL, suggesting the predominance of the Th2 response in HIV/HPV co-infected patients. However, further studies with longer follow-up will be needed to evaluate the association of IL-10 with HPV infection, CIN, and cervical cancer in WLHA.

## 1 Introduction

Acquired immunodeficiency syndrome (AIDS) was first described in 1981 (1), and since then 85.6 million people have been infected with the HIV virus and about 40.4 million have died of AIDS (2). In 2022, of the 39.0 million people living with the human immunodeficiency virus (PLHIV), 53% of them were women and girls (3). Despite the wider access to antiretroviral therapy (ART), of which 29.8 million people in December 2022 against 7.7 million in 2010 have increased the survival of this population and reduced the incidence of AIDS-associated neoplasms, the incidence of cervical cancer remains high when compared to the uninfected population (4).

In 2030, cervical cancer will account for an estimated 727,500 new cancer cases and 432,000 deaths worldwide (5). The association between human papillomavirus (HPV) and cervical cancer is already well established. While HPV is essential in the process, the infection itself is not sufficient, and a variety of cofactors can influence the development of cervical cancer. HIV is associated with increased prevalence of high-risk HPV (HR-HPV) infection and cervical intraepithelial neoplasia (CIN), which will result in the increased frequency of cervical cancer. HIV and HPV share the same transmission pathway, but the role of HIV in cervical carcinogenesis involves several mechanisms, such as inhibition of tumor suppressor genes, changes in the regulation of the cell cycle, and activation of proto-oncogenes (6).

Inflammation is a nonspecific defense mechanism to an aggressive agent, characterized by recruitment of leukocytes to the affected tissue. It aims to restore the body’s homeostasis, but if the stimulus persists, the inflammation becomes chronic, promoting an increase in cell division and the risk of mutation and neoplastic transformation (7).

Cytokines are protein molecules produced by leukocytes that play an important role in defending against infections through the cells of the immune system. Helper T cells (Th), when stimulated by antigen-presenting cells, can differentiate into several sets of effector cells, with Th1, Th2, Th17, and Treg subsets being the best known. Th1 cells are responsible for activating cellular immunity, releasing IFN-y, a potent cytokine with antiviral properties, in addition to other cytokines such as interleukin 2 (IL-2) and TNF-β. On the other hand, Th2 cells are essential for humoral immunity and the development of allergy and asthma, and Treg cells play an important role in suppressing the immune response; both of them produce the anti-inflammatory cytokine, IL-10. Lower serum concentrations of IFN-y and higher serum concentrations of IL-10 were found in women with cervical cancer or CIN, suggesting that an imbalance between Th1/Th2 and Th17/Treg cells may be responsible for the persistent HPV infection and progression to cancer (7, 8). Women living with HIV/AIDS (WLHA) also showed increased levels of IL-10 in cervical secretions compared to seronegative patients, suggesting that there is a predominance of Th2 cytokines in HIV-positive women (9). However, there is controversial data on the role of this cytokine profile on the prevalence and persistence of HR-HPV infection in this population. Despite improvements in the quality of life and increased survival with the use of ART, cervical cancer continues to be a problem in WLHA. Knowing the mechanisms involved in HPV infection can help understand other infections, in addition to being important for a more effective cervical cancer screening and prevention program. The purpose of this study is to evaluate the presence of cytokines in cervicovaginal lavage (CVL) and its association with HPV infection, HIV viral suppression, and other clinical and socio-demographic characteristics, in addition to risk factors for HR-HPV infection in WLHA followed at a referral service in Brazil.

## 2 Materials and methods

### 2.1 Study participants

This study included 106 WLHA attended at the Hospital Universitario Professor Edgard Santos (HUPES), Federal University of Bahia, Salvador, Brazil, during the period from December 2019 to April 2023.

### 2.2 Inclusion and exclusion criteria

WLHA aged 18 or older, with ability to understand and sign an informed consent form, were eligible to participate in the study. Pregnant, women who underwent hysterectomy or underwent any other surgical procedure of the uterine cervix as well as women with a history of cervical cancer or pelvic radiotherapy were excluded from the study. Women undergoing antibiotic treatment for an acute infectious process or who had recent use of vaginal antibiotics were instructed to return 30 days after the end of treatment.

### 2.3 Ethics

The study was approved by the Ethics and Research Committee of the School of Medicine of the Federal University of Bahia (approval number 2.985.561; October 27, 2018), and it was conducted according to the Declaration of Helsinki.

### 2.4 Sample collection

Sociodemographic and clinical data were obtained through a standardized questionnaire administered by the investigators during medical care.

All women underwent gynecological examination with a non-lubricated vaginal speculum and had the following samples collected: a smear from the middle third of the vaginal wall to perform the Nugent score (10), an endocervical brush for HPV genotyping, and another for screening for sexually transmitted infections (STIs). Next, a CVL was performed by gentle and repeated lavage (three times) of the cervix with sterile saline (10 mL). The fluid was allowed to accumulate in the posterior fornix, where it was then aspirated by syringe, dispensed into a sterile container, and centrifuged. The supernatant was stored at -80°C until testing (11). To minimize hormonal interference on proinflammatory cytokines’ concentrations, we scheduled the sample collection to be during the first phase of the menstrual cycle. All participants underwent colposcopy and cervical smear collection with an endocervical brush and an Ayre spatula, which was evaluated by standard Papanicolaou staining and microscopy. Colposcopy was performed at the first visit regardless of the result of cytology or HPV genotyping using the International Federation for Cervical Pathology and Colposcopy Terminology (12). Biopsy of the uterine cervix was performed when necessary.

Cytological or histological changes were treated according to the Brazilian Cervical Cancer Screening Guidelines (13).

### 2.5 Laboratory tests

#### 2.5.1 Detection of HPV, *Chlamydia trachomatis* and *Neisseria gonorrhoeae*

For HPV screening, a cervical sample was processed through a sample collection kit (Abbott), and the presence of HPV-DNA was analyzed using Alinity ^m^ PCR machine (Abbott, Wiesbaden, Germany). The Alinity ^m^ HPV test detects HPV 16, HPV 45, and HPV 18 individually and simultaneously, identifies a pool of other high-risk “A” HPV—31, 33, 52 and 58—and other high-risk “B” HPV—35, 39, 51, 56, 59, 66 and 68.

For the detection of *Chlamydia trachomatis* (CT) and *Neisseria gonorrhoeae* (NG), we used the Cobas^®^ 5800 PCR (Roche, Mannheim, Germany). PCR tests were processed in the Public Health Central Laboratory Professor Gonçalo Moniz (LACEN—Bahia, Brazil).

#### 2.5.2 Measurement of cytokines in cervicovaginal lavage

Cervicovaginal secretion supernatant samples were tested for levels of IFN-γ-induced protein 10 (IP-10), granulocyte-macrophage colony-stimulating factor (GM-CSF), macrophage inflammatory protein 1α (MIP-1α), IL-1β, IL-2, IL-4, IL-6, IL-10, IFN-γ, and TNF-α using the ProcartaPlex Custom Multiplex immune-bead assay kit (10 PPX-10-MX2W9NX Invitrogen Plex, Vienna, Austria) according to the manufacturer’s instructions. Briefly, a total of 25 µL of beads was placed into a 96-well plate. The beads were then washed once with the wash buffer provided by the manufacturer, and the samples were transferred to each well.

Serial dilution for standards was performed and placed in the appropriated wells. The plate was incubated overnight at 4°C. The beads were washed twice with the wash buffer, and the detection antibody was added and incubated for 30 min. The beads were washed again followed by the addition of 50 μL of streptavidin–phycoerythrin (PE) to each well. After washing the beads two times more with the wash buffer, they were resuspended in the read buffer and shaken for 5  min. Results were obtained by an MAGPIX instrument (Luminex Corporation, Austin, USA) and analyzed using Luminex xPONENT® multiplex assay analysis software (v.4.2.1324.0, Luminex Corporation). A 4 PL regression formula was used to calculate the sample concentrations from the standard curves.

#### 2.5.3 Viral load and CD4+ T cell

HIV viral loads (VL) were determined in plasma using real-time PCR (Abbott Molecular, IL, USA) with a lower quantification limit of 40 copies/mL. CD4+ T cell counts were performed using flow cytometry (FACSCalibur, Becton and Dickinson, CA, USA).

### 2.6 Statistical analysis

For analysis purposes, some continuous variables were stratified as dichotomic ones as follows: age was stratified into ≤30 and >30 years old because of the recommendation for cervical cancer screening among all asymptomatic individuals with a cervix, regardless of their sexual history, with cytology alone, HR-HPV testing alone, or with both tests in women aged 30 to 65 years (14). For school-age years (< 8 and ≥8 years), we considered that elementary education (<8 years) is the first stage of basic education in Brazil and is mandatory for all children. We stratified the number of previous pregnancies in less than or equal to 3 or more than 3 because it was the median number in our sample (range, 0 to 7). The number of lifetime sexual partners (≤4 and >4) was also stratified according to the median number of sexual partners (median number, 4; range 0 to 100 partners) as reported by the participants. Plasma HIV-1 VL was stratified according to the limits for detection of the PCR tests usually used, with values above 40 copies/mL classified as detectable. The mean CD4 count was 656 ± 354 cells/mL and stratified into <500 and ≥500 cells/mm^3^ because this cutoff has been used to define normal values for this test, and it was close to the sample’s median CD4 (579 cells/mL).

In addition, because some cytokines presented with very low levels in most women, we used dichotomous variables for analysis purposes, and the cytokine levels were stratified as positive if the levels were higher than their respective cutoff IFN-γ (detection limits: 4.61 –52,754 pg/mL), IP-10 (0.82–13,980 pg/mL), GM-CSF (3.22–162,744 pg/mL), MIP-1α (1.15–725.55 pg/mL), IL-1β (0.31–34,634 pg/mL), IL-2 (2.02–95,022 pg/mL), IL- 4 (6.25–66,648 pg/mL), IL-6 (4.78–60,529 pg/mL), IL-10 (0.27–41,165 pg/mL), and TNF-α (1.73–110,858 pg/mL) or negative if the levels were below the cutoff in most samples. For cytokines with a higher detection rate (MIP-1α, IL-1β, IL-6, IL-10, and IP-10), we also calculated the median and interquartile range (IQR), as shown in **Supplementary Table S1**.

Sociodemographic and clinical data were described as frequency and proportions of variables. Associations between dichotomous variables were evaluated using Pearson’s chi-square. Fisher’s exact test was used when necessary. Continuous variables were compared using Mann–Whitney *U*-test. *P*-values lower than 0.05 were considered statistically significant. The statistical analysis was performed using SPSS, version 18.0.

## 3 Results

There were 106 WLHA included in the study, all followed up at HUPES. Clinical and socio-demographic characteristics are shown in **Table 1**.

**Table 1:** Clinical and socio-demographic characteristics of 106 women living with HIV/AIDS followed at the Hospital Universitario Professor Edgard Santos, Bahia, Brazil.

Characteristics	n (%)
Age -median (IQI)	45.5 (38.0 – 53.0)
<8 schooling years	91 (85.8)
History of drug use	9 (8.5)
Regular alcohol use	32 (30.2)
Smoking	7 (6.6)
>3 lifetime sexual partners	65 (61.3)
COC use	8 (7.5)
DMPA use	6 (5.7)
>3 pregnancies	31 (29.2)
Premenopausal status	72 (67.9)
Viral load >40 cp/mL	13 (12.3)
CD4 <500 cells/mm^3^	34 (32.1)
High-risk HPV	24 (22.6)
Vaginosis by Nugent score	33 (32.0)^a^
Altered Pap test	6 (5.7)
Altered colposcopy	6 (5.7)

COC, combined oral contraceptive; DMPA, depot medroxyprogesterone acetate.

a Three samples were considered unsatisfactory for Nugent score; therefore, the population studied for vaginosis included only 103 women.

In this study, 22.6% (24/106) of WLHA were infected with HR-HPV, 4.2% (1/24) with HPV 16, 8.3% (2/24) with HPV 18, 4.2% (1/24) with HPV 45, and 79.2% (19/24) with another HR-HPV. Of the 24 WLHA infected with RH-HPV, 4.2% (1/24) were infected with another HR-HPV in addition to HPV 16 and HPV 45.

With regard to sexual activity, 46.7% (49/105) of the WLHA did not report an active sexual life in the 3 months prior to the consultation. Of the patients infected with HR-HPV, 29.2% (7/24) and 16.7% (4/24) did not report an active sexual life in the last 12 and 24 months before the consultation, respectively.

Only one woman was not on regular ART (0.9%). Dolutegravir-based ART was used by 56.6% (60/106) of women, while efavirenz was used by 22.6% (24/106) and other ART regimens were used by 19.8% (21/106) of them.

The presence of cytokines in CVL of WLHA and their main associations are described in **Table 2** and **Supplementary Table S1**. VL and CD4 were not associated with the studied cytokines in this group, except TNF-α, which showed a positive association with CD4 ≥500 cells (*p* = 0.017).

**Table 2:** Association between cytokines in cervicovaginal lavage and menopausal status, vaginosis, high-risk HPV, and altered Pap test of 106 women living with HIV/AIDS followed at the Hospital Universitario Professor Edgard Santos, Bahia, Brazil.

Premenopausal				Vaginosis^a^			High-risk HPV			Altered Pap Test			
Cytokines	Yes n=72 (%)	No n=34 (%)	p	Yes n=33 (%)	No n=70 (%)	p	Yes n=24 (%)	No n=82 (%)	p	Yes n=6 (%)	No n=100 (%)	p
**IP-10**	42 (58.3)	11 (32.4)	0.01	8 (24.2)	43 (61.4)	<0.001	14 (58.3)	39 (47.6)	0.24	6 (100.0)	47 (47.0)	0.01
**GM-CSF**	50 (69.4)	21 (61.8)	0.28	22 (66.7)	48 (68.6)	0.51	17 (70.8)	54 (65.9)	0.42	3 (50.0)	68 (68.0)	0.31
**MIP- α**	32 (44.4)	21 (61.2)	0.07	14 (42.4)	36 (51.4)	0.26	13 (54.2)	40 (48.8)	0.41	2 (33.3)	51 (51.0)	0.33
**IL-1β**	34 (47.2)	17 (50.0)	0.47	22 (66.7)	26 (37.1)	0.005	13 (54.2)	38 (46.3)	0.32	2 (33.3)	49 (49.0)	0.37
**IL-2**	1 (1.4)	0 (0.0)	0.67	0 (0.0)	1 (1.4)	0.68	0 (0.0)	1 (1.2)	0.77	0 (0.0)	1 (1.0)	0.94
**IL-4**	9 (12.5)	5 (14.7)	0.48	6 (18.2)	8 (11.4)	0.26	3 (12.5)	11 (13.4)	0.60	0 (0.0)	14 (14.0)	0.41
**IL-6**	37 (51.4)	9 (26.5)	0.01	17 (51.5)	26 (37.1)	0.12	13 (54.2)	33 (40.2)	0.16	3 (50.0)	43 (43.0)	0.52
**IL-10**	30 (41.7)	19 (55.9)	0.12	21 (63.6)	26 (37.1)	0.01	16 (66.7)	33 (40.2)	0.02	2 (33.3)	47 (47.0)	0.41
**IFN--γ**	45 (62.5)	17 (50.0)	0.15	13 (39.4)	48 (68.6)	0.005	13 (54.2)	49 (59.8)	0.39	3 (50.0)	59 (59.0)	0.48
**TNF- α**	12 (16.7)	3 (8.8)	0.22	5 (15.2)	10 (14.3)	0.56	4 (16.7)	11 (13.4)	0.45	1 (16.7)	68 (68.0)	0.61

IP-10, IFN-γ induced protein 10; GM-CSF, granulocyte-macrophage colony-stimulating factor; MIP-1, macrophage inflammatory protein 1; IL, interleukin; IFN, interferon; TNF, tumor necrosis factor.

a Three samples were considered unsatisfactory for Nugent score, therefore the population studied for vaginosis included only 103 women.

**Table 3** summarizes the risk factors significantly associated with HR-HPV infection of WLHA. Altered Pap test was found in six women: three with ASC-US, one with low-grade intraepithelial lesion (LSIL), one with high-grade intraepithelial lesion (HSIL), and one with atypical squamous cells cannot exclude high-grade squamous intraepithelial lesion (ASC-H). To date, we have had only one invasive cervical cancer that was confirmed by anatomopathological examination.

**Table 3:** Risk factors associated with high-risk HPV infection in 106 women living with HIV/AIDS followed at the Hospital Universitario Professor Edgard Santos, Bahia, Brazil.

		High-risk HPV				p
		Yes		No		
		n	%	n	%	
Menopause	Yes	3	8.8%	31	91.2%	0.015
	No	21	29.2%	51	70.8%	
Vaginosis^a^	Yes	13	39.4%	20	60.6%	0.009
	No	11	15.7%	59	84.3%	
Altered Pap Test	Yes	4	66.7%	2	33.3%	0.023
	No	20	20.0%	80	80.0%	
CD4<500 cells	Yes	14	41.2%	20	58.8%	0.002
	No	10	13.9%	62	86.1%	

a Three samples were considered unsatisfactory for Nugent score, therefore the population studied for vaginosis included only 103 women.

Age, years of schooling, regular use of alcohol, combined oral contraceptive pill (COC) use, depot medroxyprogesterone acetate (DMPA) use, numbers of pregnancies and lifetime partners, VL, altered colposcopy, reported sexual intercourse within less than 90 days and less than 12 months, ART, condom use, and CT were not associated with HR-HPV in the population studied.

Patients with HR-HPV were invited for a new collection after 12 months, but only 83.3% (20/24) attended the scheduled medical visit. Of these, 65% (13/20) remained positive for HR-HPV, but due to the lack of individualization of all HPV subtypes, it was only possible to characterize the persistence of infection in two patients, one infected with HPV 16 and the other with HPV 45.

## 4 Discussion

In the current work, HR-HPV infection was significantly associated with the detection of IL-10 in CVL of WLHA. This study confirms the findings published by Fernandes et al. (15) that describe an increase of IL-10 in WLHA coinfected with HPV when compared to those infected with HIV alone but which are in conflict with those of Kriek et al. (16) who found no difference in IL-6, IL-10, and IFN-γ levels using cervical cytobrush supernatants in a cohort study with 93 WLHA. However, the median level of IL-10 was not different for HR-HPV (median: -1.02; IQR: 0.56–1.34) and non-HR-HPV groups (median: 0.61; IQR: 0.45–1.15; *p* = 0.071).

Viral load and CD4 were also not associated with cytokine detection, in accordance with a previous study that found no correlation between peripheral CD4 count and cytokine using cytokine mRNA optical density in cervical biopsies (17).

We found a statistically significant association between the presence of IL-6 in CVL and non-menopause WLHA (*p* = 0.013). Previous studies have shown increased IL-6 concentrations in HIV-infected women compared to HIV-negative ones (9, 16) in cervical HPV infection compared with negative HPV and in abnormal cervical cytology compared with normal cervical cytology (9), but it was not related to hormonal status. Although IL-6 is a pro-inflammatory cytokine and its levels are very high in processes involving an inflammatory response (including autoimmune diseases like rheumatoid arthritis), IL-6 can also exhibit anti-inflammatory effects via the membrane-bound IL-6R (18).

Although the hormonal production of non-menopausal women influences the maintenance of genital health through the conversion of glycogen into lactic acid under the action of local bacterial flora (19), STIs are more frequent in the young population (20, 21), including HR-HPV in young WLHA (22, 23), which could justify the increase in IL-6 in the younger population.

Significantly higher levels of IP-10 were detected in pre-menopausal women (*p* = 0.011) and altered Pap test (*p* = 0.013). Elevated plasma levels of IP-10 have been described in HIV-infected individuals and in individuals who are co-infected with HIV and hepatitis C, tuberculosis, or cryptosporidiosis when compared with HIV mono-infected individuals and are associated with disease progression (24). Although higher genital concentrations of IP-10 were found in WLHA than in HIV-negative individuals by Kriek et al. (16), the association observed in our study could also be explained by the presence of subclinical HPV evidenced in the altered Pap tests.

Vaginosis is characterized by the loss of *Lactobacillus* dominance and colonization by anaerobic and aerobic species. Although it is not associated with typical signs and symptoms of an inflammatory process, it has been associated with an increased risk of STIs, including HIV and HPV, and slower clearance of HPV (25, 26). In the present work, we detected a significant association between vaginosis and WLHA infected with HR-HPV (*p* = 0.009), confirming previous findings. Many studies have measured cervicovaginal cytokine levels in vaginosis in HIV-uninfected women with discordant results. In most studies, IL-1β was associated with vaginosis, but the same did not occur with IL-6, IL-10, INF-γ, and other cytokines (27). We found 32.0% (33/103) of vaginosis in the studied population and a significant association with IL-1β, IL-10, INF-γ, and IP-10. In our study, the levels of these cytokines were significantly higher than those found in women without vaginosis, except for IFN-γ and IP-10 that were significantly lower in WLHA with vaginosis. These findings suggest that IFN-γ and IP-10 are important in the protection against vaginosis in such population, as they were detected in increased levels in women without vaginosis.

We also detected HR-HPV infection in 22.6% (24/106) of WLHA. Such prevalence was lower than that found in other works in the northeast region of Brazil (22, 28), probably due to the specific characteristics of our population, like a higher median age (45.5 years).

A lower CD4 cell count in WLHA has been associated with a higher prevalence and decreased clearance of HR-HPV (22, 29). Our findings confirm such association with the detection of HR-HPV in 14/34 (41.2%) WLHA with CD4 <500 cells/mL (*p* = 0.002), while HR-HPV was detected in only 10/72 (13.9%) of those presenting with a CD4 cell count ≥500 cells/mL, suggesting that WLHA with CD4 <500 should remain under close surveillance for cervical cancer.

The main limitation of this study was the sample size, although few studies have a large number of samples, such as those of Kriek et al. (16) with 93 WLHA and Buckley et al. (30) with 128. The HPV test used does not detect all types of HPV individually, and tests for other STIs such as *Trichomonas vaginalis* and herpes simplex virus were not available, which could alter the detection of cytokines.

Given the lack of a well-established methodology for measuring cytokines in CVL and microbial and host diversity, comparisons between studies should be carefully evaluated. Viral load was not available in cervical fluid because plasma viral load is the strongest predictor of CVL fluid HIV-1 RNA detection; it is unlikely to find the virus in the genital tract when the plasma viral load is undetectable (31).

Understanding how the local immune response occurs in WLHA is important not only in preventing persistent HPV infection but also in the management of other STIs. Our study detected a statistically significant association between IL-10 and HPV infections, suggesting the predominance of the Th2 response in HIV/HPV co-infected patients. In addition, we detected a statistically significant association between IL-6 and IP-10 levels and premenopausal women, while higher levels of IL-1β and IL-10 and lower levels of IP-10 and IFN-γ were associated with vaginosis in this population, reinforcing the multiplicity of factors involved in the local immune response. Further studies with longer follow-up will be necessary to evaluate the association of IL-10 with the persistence of HPV infection, CIN, and cervical cancer in WLHA.

## Data availability statement

The original contributions presented in the study are included in the article/**Supplementary Material**. Further inquiries can be directed to the corresponding author.

## Ethics statement

The studies involving humans were approved by the Comitê de Ética em Pesquisa: Faculdade de Medicina da Bahia(FMB) da Universida Federal da Bahia. The studies were conducted in accordance with the local legislation and institutional requirements. The participants provided their written informed consent to participate in this study.

## Author contributions

SS: Conceptualization, Formal Analysis, Investigation, Methodology, Project administration, Writing – original draft, Writing – review & editing. EN: Formal Analysis, Investigation, Methodology, Writing – original draft, Writing – review & editing. FD: Investigation, Methodology, Writing – original draft, Writing – review & editing. CF: Methodology, Supervision, Writing – original draft, Writing – review & editing. CA: Methodology, Writing – original draft, Writing – review & editing. AA: Methodology, Writing – original draft, Writing – review & editing. CB: Conceptualization, Methodology, Project administration, Resources, Supervision, Writing – original draft, Writing – review & editing.
