# Supplementary material for: Detection of cytokines in cervicovaginal lavage in HIV-infected women and its association with high-risk human papillomavirus
Source: Front Immunol. 2024 Jun 28;15:1416204. doi: 10.3389/fimmu.2024.1416204 (PMC11239429; doi:10.3389/fimmu.2024.1416204)
Supplement: Supplementary file 1 [file Table_1.docx]

Supplementary Material

**Supplementary Table 1 – Median levels of MIP-1α, IL-1β, IL-6, IL-10 and IP-10 in cervicovaginal lavage according to menopausal status, vaginosis, high-risk HPV and altered Pap Test of 106 women living with HIV/AIDS followed at the Hospital Universitario Professor Edgard Santos, Bahia, Brazil.**

|  | Premenopausal Status (n=72) | | | Vaginosis*  (n=33) | | | High-risk HPV (n=24) | | | Altered Pap Test  (n=6) | | |
| --- | --- | --- | --- | --- | --- | --- | --- | --- | --- | --- | --- | --- |
| **Cytokine** | yes | no | p | yes | no | p | yes | no | p | yes | no | p |
| **MIP-1α** | .11 (.00 -.49) | .31 (.00 -1.36) | .031 | .12 (00 - .44) | .18 (.00 - .78) | .482 | .19 (.00 -.82) | .13 (.00 -.55) | .675 | .06 (.00-.19) | .17(.00-.65) | .487 |
| **IL-1β** | 18.28 (3.80-57.50) | 19.59 (5.41-57.54) | .951 | 32.32 (13.32 – 134.77) | 11.60 (3.20 – 34.83) | .003 | 27.61 (6.68 – 121.36) | 14.25 (3.39 – 43.54) | .103 | 13.63 (6.94-263.45) | 18.28 (4.29-56.37) | .697 |
| **IL-6** | 21.21 (10.50 –40.12) | 9.09 (5.27-19.07) | .001 | 19.07 (7.18 - 36.52) | 12.83 (7.18 – 28.16) | .278 | 29.30 (9.09 – 55.58) | 14.65 (7.18 – 27.36) | .082 | 19.70 (10.97-35.78) | 16.45 (7.18-32.42) | .583 |
| **IL-10** | .61 (.45 1.12) | .79 (.45 1.53) | .437 | .84 (.56 – 1.15) | .59 (.45 – 1.40) | .317 | 1.02 (.56 – 1.34) | .61 (.45 – 1.15) | .071 | .51 (.45-1.02) | .67(.45-1.27) | .463 |
| **IP-10** | 24.27 (8.39 – 93.03) | 10.09 (1.40-28.63) | .007 | 13.41 2.22 – 22.24 | 26.75 (8.13 – 89.01) | .004 | 24.27 (3.87 – 63.27) | 20.91 (6.39 – 69.60) | .792 | 36.86 (23.69-79.30) | 20.91 (5.79-65.94) | .116 |

MIP-1: macrophage inflammatory protein 1; IL: interleukin; IP-10: IFN-γ induced protein 10; GM-CSF: granulocyte-macrophage colony-stimulating factor. * Three samples were considered unsatisfactory for Nugent score, therefore the population studied for vaginosis included only 103 women. All values are expressed in pg/mL.
